# Crystal structure of tri­aqua­(2,6-di­methyl­pyrazine-κ*N* ^4^)bis­(thio­cyanato-κ*N*)manganese(II) 2,5-di­methyl­pyrazine disolvate

**DOI:** 10.1107/S2056989015020769

**Published:** 2015-11-18

**Authors:** Stefan Suckert, Susanne Wöhlert, Inke Jess, Christian Näther

**Affiliations:** a Institut für Anorganische Chemie, Christian-Albrechts-Universität Kiel, Max-Eyth-Strasse 2, 24118 Kiel, Germany

**Keywords:** crystal structure, coordination complex, manganese(II), hydrogen bonding

## Abstract

In the crystal structure of the title complex, [Mn(NCS)_2_(C_6_H_8_N_2_)(H_2_O)_3_]·2C_6_H_8_N_2_, the Mn^II^ cation is coordinated by two terminally *N*-bonded thio­cyanate anions, three water mol­ecules and one 2,6-di­methyl­pyrazine ligand within a slightly distorted N_3_O_3_ octa­hedral geometry; the entire complex mol­ecule is generated by the application of a twofold rotation axis. The asymmetric unit also contains an uncoordinating 2,5-di­methyl­pyrazine ligand in a general position. Obviously, the coordination to the 2,6-di­methyl­pyrazine ligand is preferred because coordination to the 2,5-di­methyl­pyrazine is hindered due to the bulky methyl group proximate to the N atom. The discrete complexes are linked by water-O—H⋯N(2,6-di­methyl­pyzazine/2,5-di­methyl­pyza­zine) hydrogen bonding, forming a three-dimensional network. In the crystal, mol­ecules are arranged in a way that cavities are formed in which unspecified, disordered solvent molecules reside. These were modelled employing the SQUEEZE routine in *PLATON* [Spek (2015 ▸). *Acta Cryst*. C**71**, 9–18]. The composition of the unit cell does not take into account the presence of the unspecified solvent.

## Related literature   

For structures with metal thio­cyanates and 2,5-di­methyl­pyrazine or 2,6-di­methyl­pyrazine, see: Otieno *et al.* (2003 ▸); Mahmoudi & Morsali (2009 ▸).
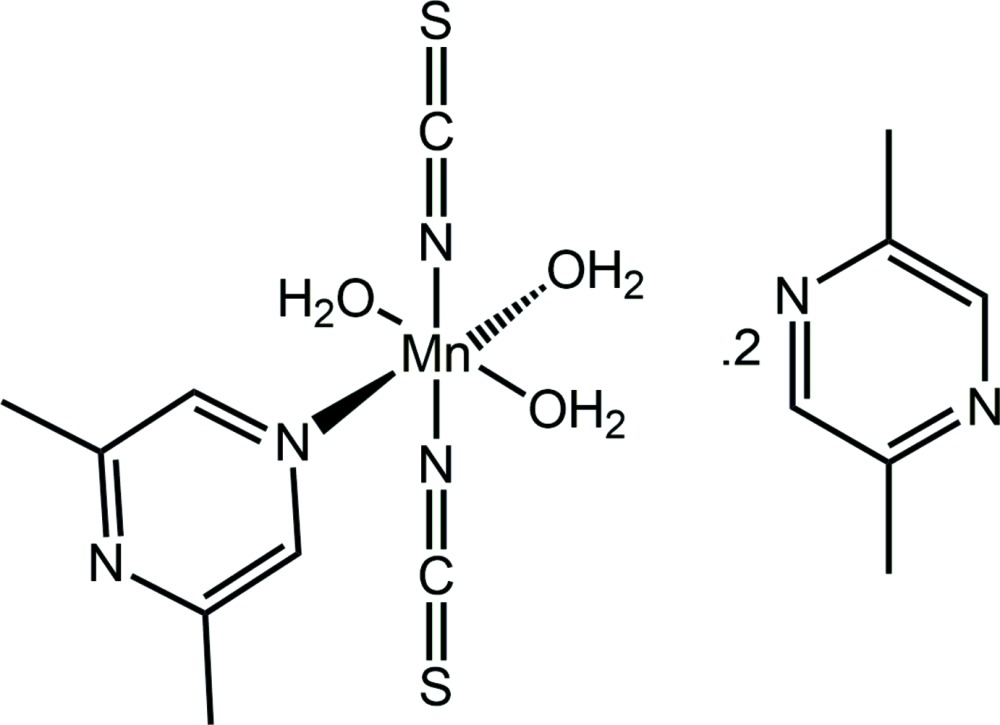


## Experimental   

### Crystal data  

[Mn(NCS)_2_(C_6_H_8_N_2_)(H_2_O)_3_]·2C_6_H_8_N_2_
*M* *_r_* = 549.58
Monoclinic, 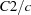

*a* = 15.365 (1) Å
*b* = 27.9630 (14) Å
*c* = 7.0816 (5) Å
β = 93.59 (3)°
*V* = 3036.6 (3) Å^3^
*Z* = 4
Mo *K*α radiation
μ = 0.60 mm^−1^
*T* = 200 K
0.30 × 0.23 × 0.13 mm

### Data collection  

Stoe IPDS-1 diffractometer
Absorption correction: numerical (*X-SHAPE* and *X-RED32*; Stoe, 2008 ▸) *T*_min_ = 0.839, *T*_max_ = 0.921
12096 measured reflections
3674 independent reflections
2928 reflections with *I* > 2σ(*I*)
*R*_int_ = 0.034

### Refinement  

*R*[*F* ^2^ > 2σ(*F* ^2^)] = 0.046
*wR*(*F* ^2^) = 0.137
*S* = 1.07
3674 reflections
160 parameters
H-atom parameters constrained
Δρ_max_ = 0.56 e Å^−3^
Δρ_min_ = −0.45 e Å^−3^

### 

Data collection: *X-AREA* (Stoe, 2008 ▸); cell refinement: *X-AREA*; data reduction: *X-AREA*; program(s) used to solve structure: *SHELXS97* (Sheldrick, 2008 ▸); program(s) used to refine structure: *SHELXL2013* (Sheldrick, 2015 ▸); molecular graphics: *XP* in *SHELXTL* (Sheldrick, 2008 ▸) and *DIAMOND* (Brandenburg, 1999 ▸); software used to prepare material for publication: *publCIF* (Westrip, 2010 ▸).

## Supplementary Material

Crystal structure: contains datablock(s) I, global. DOI: 10.1107/S2056989015020769/tk5403sup1.cif

**Fig. 1.**
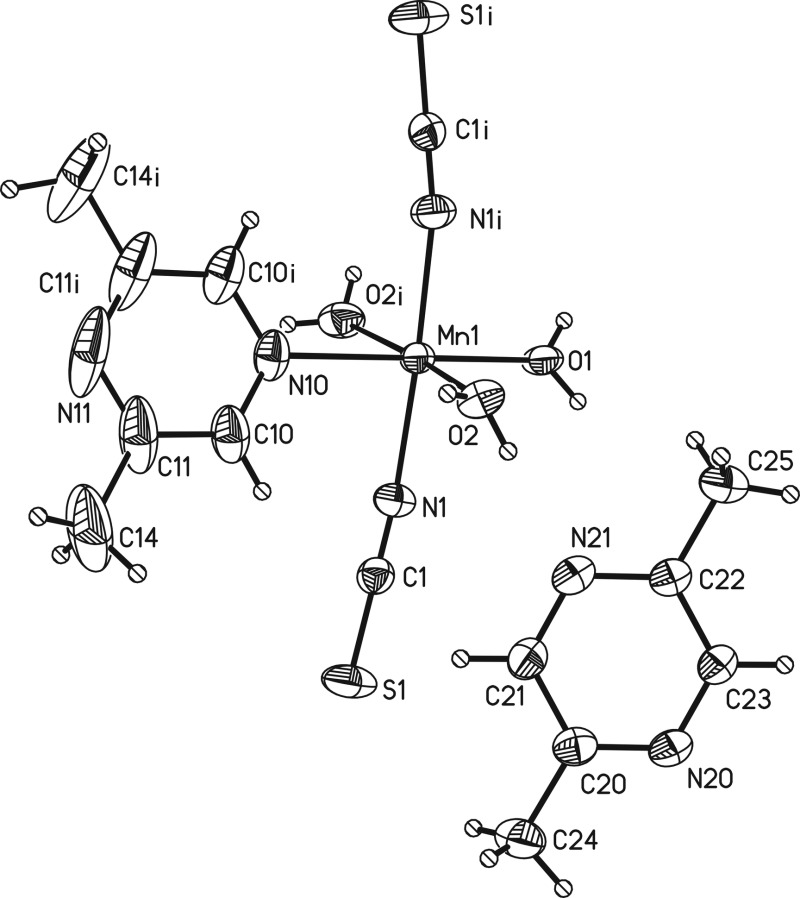
The molecule structures of the complex molecule (located on a 2-fold axis) and solvent molecule (full weight) in the title compound with labelling and displacement ellipsoids drawn at the 50% probability level. [Symmetry code: (i) = -x + 1, y, -z + 3/2].

**Fig. 2.**
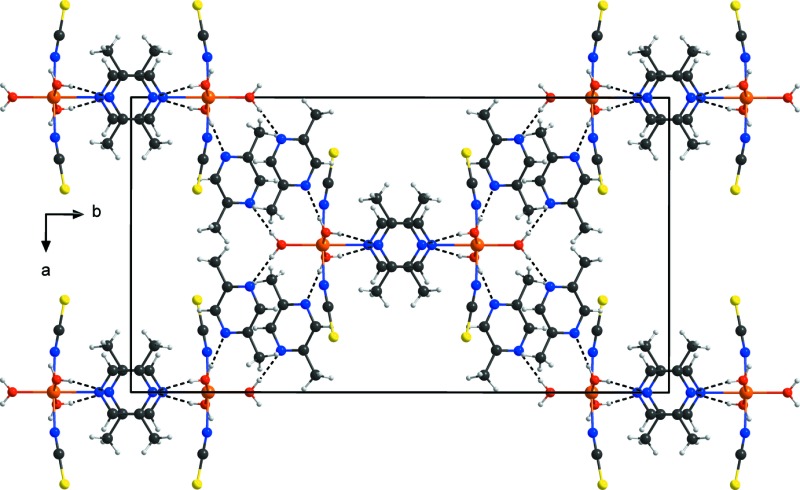
Part of the crystal structure of the title compound viewed along the c axis. Hydrogen bonding is shown as dashed lines.

Click here for additional data file.x y z . DOI: 10.1107/S2056989015020769/tk5403fig1.tif
The mol­ecule structures of the complex mol­ecule (located on a 2-fold axis) and solvent mol­ecule (full weight) in the title compound with labelling and displacement ellipsoids drawn at the 50% probability level. [Symmetry code: (i) = −*x* + 1, *y*, −*z* + 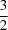].

c . DOI: 10.1107/S2056989015020769/tk5403fig2.tif
Part of the crystal structure of the title compound viewed along the *c* axis. Hydrogen bonding is shown as dashed lines.

CCDC reference: 1434562

Additional supporting information:  crystallographic information; 3D view; checkCIF report

## Figures and Tables

**Table 1:** Hydrogen-bond geometry (Å, °)

*D*—H⋯*A*	*D*—H	H⋯*A*	*D*⋯*A*	*D*—H⋯*A*
O1—H1*O*1⋯N20^i^	0.82	1.97	2.790 (2)	177
O2—H1*O*2⋯N21	0.82	1.95	2.769 (2)	179
O2—H2*O*2⋯N11^ii^	0.82	2.33	3.138 (3)	170

Symmetry codes: (i) 
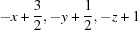
; (ii) 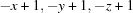.

## supplementary crystallographic information

### S1. Synthesis and crystallization

MnSO_4_.H_2_O was purchased from Merck and 2,5-di­methyl­pyrazine (97%) and Ba(NCS)_2_.3H_2_O were purchased from Alfa Aesar. Mn(NCS)_2_ was synthesized by stirring 17.97 g (58.44 mmol) Ba(NCS)_2_.3H_2_O and 9.88 g (58.44 mmol) MnSO_4_.H_2_O in H_2_O (300 mL) at RT for 3 h. The white residue of BaSO_4_ was filtered off and the solvent removed with a rotary evaporator. The title compound was prepared by the reaction of Mn(NCS)_2_. H_2_O (60.1 mg, 0.25 mmol) and 2,5-di­methyl­pyrazine (108.0 µL, 1.00 mmol) in water (1.0 mL) at RT. After few days, yellow blocks of the title compound were obtained, that contains 2,6-di­methyl­pyrazine in addition anothe rmaterial. Later, it was found that the commercially available 2,5-di­methyl­pyrazine contains about 3% of 2,6-di­methyl­pyrazine as a contamination.

### S2. Refinement

The C—H H atoms were positioned with idealized geometry and were refined isotropically with *U*_eq_(H) = 1.2 *U*_eq_(C) using a riding model with C—H = 0.95 Å for aromatic H atoms and *U*_eq_(H) = 1.5 *U*_eq_(C) and C—H = 0.98 Å for methyl H atoms. The methyl H atoms were allowed to rotate but not to tip. After refinement there is some residual electron density indicating a disordered N-donor ligand that is located on a center of inversion. As no reasonabe model was found and the identity of this moelcule is unknown the data were modelled for disordered solvent using the SQUEEZE routine in PLATON (Spek, 2015).

### Crystal data

**Table:**

[Mn(NCS)_2_(C_6_H_8_N_2_)(H_2_O)_3_]·2C_6_H_8_N_2_	*F*(000) = 1148
*M**_r_* = 549.58	*D*_x_ = 1.202 Mg m^−^^3^
Monoclinic, *C*2/*c*	Mo *K*α radiation, λ = 0.71073 Å
*a* = 15.365 (1) Å	Cell parameters from 12096 reflections
*b* = 27.9630 (14) Å	θ = 2.9–28.1°
*c* = 7.0816 (5) Å	µ = 0.60 mm^−^^1^
β = 93.59 (3)°	*T* = 200 K
*V* = 3036.6 (3) Å^3^	Block, yellow
*Z* = 4	0.30 × 0.23 × 0.13 mm

### Data collection

**Table:**

Stoe IPDS-1 diffractometer	2928 reflections with *I* > 2σ(*I*)
Radiation source: fine-focus sealed tube	*R*_int_ = 0.034
phi–scans	θ_max_ = 28.1°, θ_min_ = 2.9°
Absorption correction: numerical (*X-SHAPE* and *X-RED32*; Stoe, 2008)	*h* = −20→20
*T*_min_ = 0.839, *T*_max_ = 0.921	*k* = −36→33
12096 measured reflections	*l* = −9→9
3674 independent reflections	

### Refinement

**Table:**

Refinement on *F*^2^	Hydrogen site location: mixed
Least-squares matrix: full	H-atom parameters constrained
*R*[*F*^2^ > 2σ(*F*^2^)] = 0.046	*w* = 1/[σ^2^(*F*_o_^2^) + (0.0893*P*)^2^ + 0.7561*P*] where *P* = (*F*_o_^2^ + 2*F*_c_^2^)/3
*wR*(*F*^2^) = 0.137	(Δ/σ)_max_ = 0.001
*S* = 1.07	Δρ_max_ = 0.56 e Å^−^^3^
3674 reflections	Δρ_min_ = −0.45 e Å^−^^3^
160 parameters	Extinction correction: *SHELXL2013* (Sheldrick, 2015), Fc^*^=kFc[1+0.001xFc^2^λ^3^/sin(2θ)]^-1/4^
0 restraints	Extinction coefficient: 0.0112 (14)

### Special details

**Table:**

Geometry. All esds (except the esd in the dihedral angle between two l.s. planes) are estimated using the full covariance matrix. The cell esds are taken into account individually in the estimation of esds in distances, angles and torsion angles; correlations between esds in cell parameters are only used when they are defined by crystal symmetry. An approximate (isotropic) treatment of cell esds is used for estimating esds involving l.s. planes.

### Fractional atomic coordinates and isotropic or equivalent isotropic displacement parameters (Å^2^)

**Table:**

	*x*	*y*	*z*	*U*_iso_*/*U*_eq_	
Mn1	0.5000	0.35637 (2)	0.7500	0.02817 (16)	
N1	0.63645 (12)	0.35855 (7)	0.8519 (3)	0.0392 (4)	
C1	0.70974 (14)	0.36619 (7)	0.8897 (3)	0.0320 (4)	
S1	0.81250 (4)	0.37677 (3)	0.94387 (10)	0.0516 (2)	
N10	0.5000	0.44106 (9)	0.7500	0.0384 (6)	
C10	0.57337 (19)	0.46610 (9)	0.7441 (4)	0.0466 (6)	
H10	0.6271	0.4495	0.7390	0.056*	
C11	0.5741 (3)	0.51597 (10)	0.7453 (5)	0.0701 (11)	
N11	0.5000	0.54050 (11)	0.7500	0.0908 (18)	
C14	0.6575 (3)	0.54330 (13)	0.7435 (7)	0.1057 (18)	
H14A	0.6836	0.5460	0.8729	0.159*	
H14B	0.6458	0.5753	0.6918	0.159*	
H14C	0.6979	0.5265	0.6647	0.159*	
N20	0.85869 (11)	0.27900 (7)	0.3202 (3)	0.0333 (4)	
C20	0.85722 (13)	0.32096 (8)	0.4089 (3)	0.0328 (4)	
C21	0.77715 (14)	0.34317 (8)	0.4339 (3)	0.0340 (4)	
H21	0.7770	0.3729	0.4986	0.041*	
C22	0.70304 (13)	0.28263 (7)	0.2784 (3)	0.0293 (4)	
C23	0.78238 (13)	0.26022 (7)	0.2550 (3)	0.0317 (4)	
H23	0.7825	0.2304	0.1905	0.038*	
C24	0.94162 (15)	0.34348 (10)	0.4817 (4)	0.0481 (6)	
H24A	0.9518	0.3727	0.4099	0.072*	
H24B	0.9896	0.3210	0.4668	0.072*	
H24C	0.9385	0.3514	0.6159	0.072*	
C25	0.61833 (14)	0.26149 (9)	0.2047 (3)	0.0397 (5)	
H25A	0.5932	0.2812	0.1006	0.060*	
H25B	0.5780	0.2604	0.3064	0.060*	
H25C	0.6282	0.2290	0.1588	0.060*	
N21	0.70130 (11)	0.32445 (6)	0.3712 (3)	0.0327 (4)	
O1	0.5000	0.27960 (7)	0.7500	0.0436 (6)	
H1O1	0.5402	0.2616	0.7278	0.065*	
O2	0.54166 (10)	0.36203 (6)	0.4560 (3)	0.0431 (4)	
H1O2	0.5890	0.3508	0.4324	0.065*	
H2O2	0.5375	0.3883	0.4048	0.065*	

### Atomic displacement parameters (Å^2^)

**Table:**

	*U*^11^	*U*^22^	*U*^33^	*U*^12^	*U*^13^	*U*^23^
Mn1	0.0211 (2)	0.0233 (2)	0.0402 (3)	0.000	0.00231 (16)	0.000
N1	0.0256 (8)	0.0395 (10)	0.0520 (12)	−0.0022 (7)	0.0000 (7)	−0.0025 (9)
C1	0.0309 (10)	0.0287 (9)	0.0366 (11)	0.0015 (7)	0.0034 (8)	0.0018 (8)
S1	0.0252 (3)	0.0799 (5)	0.0492 (4)	−0.0089 (3)	−0.0008 (2)	−0.0037 (3)
N10	0.0544 (16)	0.0242 (11)	0.0377 (14)	0.000	0.0107 (12)	0.000
C10	0.0690 (17)	0.0307 (11)	0.0419 (13)	−0.0106 (10)	0.0184 (12)	−0.0035 (9)
C11	0.120 (3)	0.0331 (13)	0.0631 (19)	−0.0243 (15)	0.053 (2)	−0.0122 (12)
N11	0.157 (4)	0.0227 (14)	0.103 (3)	0.000	0.095 (3)	0.000
C14	0.154 (4)	0.0514 (19)	0.121 (4)	−0.052 (2)	0.081 (3)	−0.029 (2)
N20	0.0268 (8)	0.0384 (9)	0.0348 (9)	0.0069 (7)	0.0023 (7)	0.0026 (7)
C20	0.0293 (9)	0.0389 (11)	0.0301 (10)	0.0024 (8)	0.0012 (7)	0.0045 (8)
C21	0.0346 (10)	0.0353 (10)	0.0322 (10)	0.0052 (8)	0.0030 (8)	−0.0023 (8)
C22	0.0280 (9)	0.0341 (10)	0.0260 (9)	0.0029 (7)	0.0036 (7)	0.0044 (8)
C23	0.0306 (9)	0.0320 (10)	0.0328 (10)	0.0051 (7)	0.0032 (7)	0.0004 (8)
C24	0.0314 (11)	0.0581 (15)	0.0543 (15)	−0.0061 (10)	−0.0010 (10)	−0.0051 (12)
C25	0.0289 (10)	0.0502 (13)	0.0396 (12)	−0.0025 (9)	−0.0009 (8)	0.0023 (10)
N21	0.0299 (8)	0.0384 (9)	0.0302 (9)	0.0072 (7)	0.0043 (6)	0.0019 (7)
O1	0.0194 (9)	0.0231 (10)	0.0887 (19)	0.000	0.0056 (10)	0.000
O2	0.0298 (8)	0.0495 (10)	0.0508 (10)	0.0038 (6)	0.0098 (7)	−0.0048 (8)

### Geometric parameters (Å, º)

**Table:**

Mn1—O1	2.147 (2)	C11—N11	1.331 (4)
Mn1—N1^i^	2.175 (2)	C11—C14	1.493 (5)
Mn1—N1	2.175 (2)	N11—C11^i^	1.331 (4)
Mn1—O2^i^	2.2216 (18)	N20—C20	1.332 (3)
Mn1—O2	2.2216 (18)	N20—C23	1.340 (3)
Mn1—N10	2.368 (2)	C20—C21	1.399 (3)
N1—C1	1.161 (3)	C20—C24	1.504 (3)
C1—S1	1.628 (2)	C21—N21	1.328 (3)
N10—C10	1.330 (3)	C22—N21	1.343 (3)
N10—C10^i^	1.330 (3)	C22—C23	1.390 (3)
C10—C11	1.395 (4)	C22—C25	1.493 (3)
			
O1—Mn1—N1^i^	91.61 (5)	C10—N10—Mn1	121.76 (15)
O1—Mn1—N1	91.61 (5)	C10^i^—N10—Mn1	121.77 (15)
N1^i^—Mn1—N1	176.79 (10)	N10—C10—C11	122.2 (3)
O1—Mn1—O2^i^	94.09 (5)	N11—C11—C10	120.6 (3)
N1^i^—Mn1—O2^i^	88.89 (8)	N11—C11—C14	118.2 (3)
N1—Mn1—O2^i^	90.88 (8)	C10—C11—C14	121.2 (4)
O1—Mn1—O2	94.09 (5)	C11—N11—C11^i^	118.0 (3)
N1^i^—Mn1—O2	90.88 (8)	C20—N20—C23	117.86 (17)
N1—Mn1—O2	88.89 (8)	N20—C20—C21	119.46 (19)
O2^i^—Mn1—O2	171.82 (9)	N20—C20—C24	119.41 (19)
O1—Mn1—N10	180.0	C21—C20—C24	121.1 (2)
N1^i^—Mn1—N10	88.39 (5)	N21—C21—C20	122.9 (2)
N1—Mn1—N10	88.39 (5)	N21—C22—C23	119.70 (19)
O2^i^—Mn1—N10	85.91 (5)	N21—C22—C25	118.18 (18)
O2—Mn1—N10	85.91 (5)	C23—C22—C25	122.12 (19)
C1—N1—Mn1	169.32 (19)	N20—C23—C22	122.52 (19)
N1—C1—S1	179.7 (2)	C21—N21—C22	117.57 (17)
C10—N10—C10^i^	116.5 (3)		

Symmetry code: (i) −*x*+1, *y*, −*z*+3/2.

### Hydrogen-bond geometry (Å, º)

**Table:**

*D*—H···*A*	*D*—H	H···*A*	*D*···*A*	*D*—H···*A*
O1—H1*O*1···N20^ii^	0.82	1.97	2.790 (2)	177
O2—H1*O*2···N21	0.82	1.95	2.769 (2)	179
O2—H2*O*2···N11^iii^	0.82	2.33	3.138 (3)	170

Symmetry codes: (ii) −*x*+3/2, −*y*+1/2, −*z*+1; (iii) −*x*+1, −*y*+1, −*z*+1.

## References

[bb1] Brandenburg, K. (1999). *DIAMOND*. Crystal Impact GbR, Bonn, Germany.

[bb2] Mahmoudi, G. & Morsali, A. (2009). *CrystEngComm*, **11**, 1868–1879.

[bb3] Otieno, T., Blanton, J. R., Lanham, K. J. & Parkin, S. (2003). *J. Chem. Crystallogr.* **33**, 335–339.

[bb4] Sheldrick, G. M. (2008). *Acta Cryst.* A**64**, 112–122. 10.1107/S0108767307043930 18156677

[bb5] Sheldrick, G. M. (2015). *Acta Cryst.* C**71**, 3–8.

[bb6] Spek, A. L. (2015). *Acta Cryst.* C**71**, 9–18. 10.1107/S2053229614024929 25567569

[bb7] Stoe (2008). *X-AREA*, *X-RED32* and *X-SHAPE*. Stoe & Cie, Darmstadt, Germany.

[bb8] Westrip, S. P. (2010). *J. Appl. Cryst.* **43**, 920–925.

