# Supplementary figures and images for: Crystal structure of tri­aqua­(2,6-di­methyl­pyrazine-κN 4)bis­(thio­cyanato-κN)manganese(II) 2,5-di­methyl­pyrazine disolvate
Source: Acta Crystallogr E Crystallogr Commun. 2015 Nov 18;71(Pt 12):m223–4. doi: 10.1107/S2056989015020769 (PMC4719844; doi:10.1107/S2056989015020769)

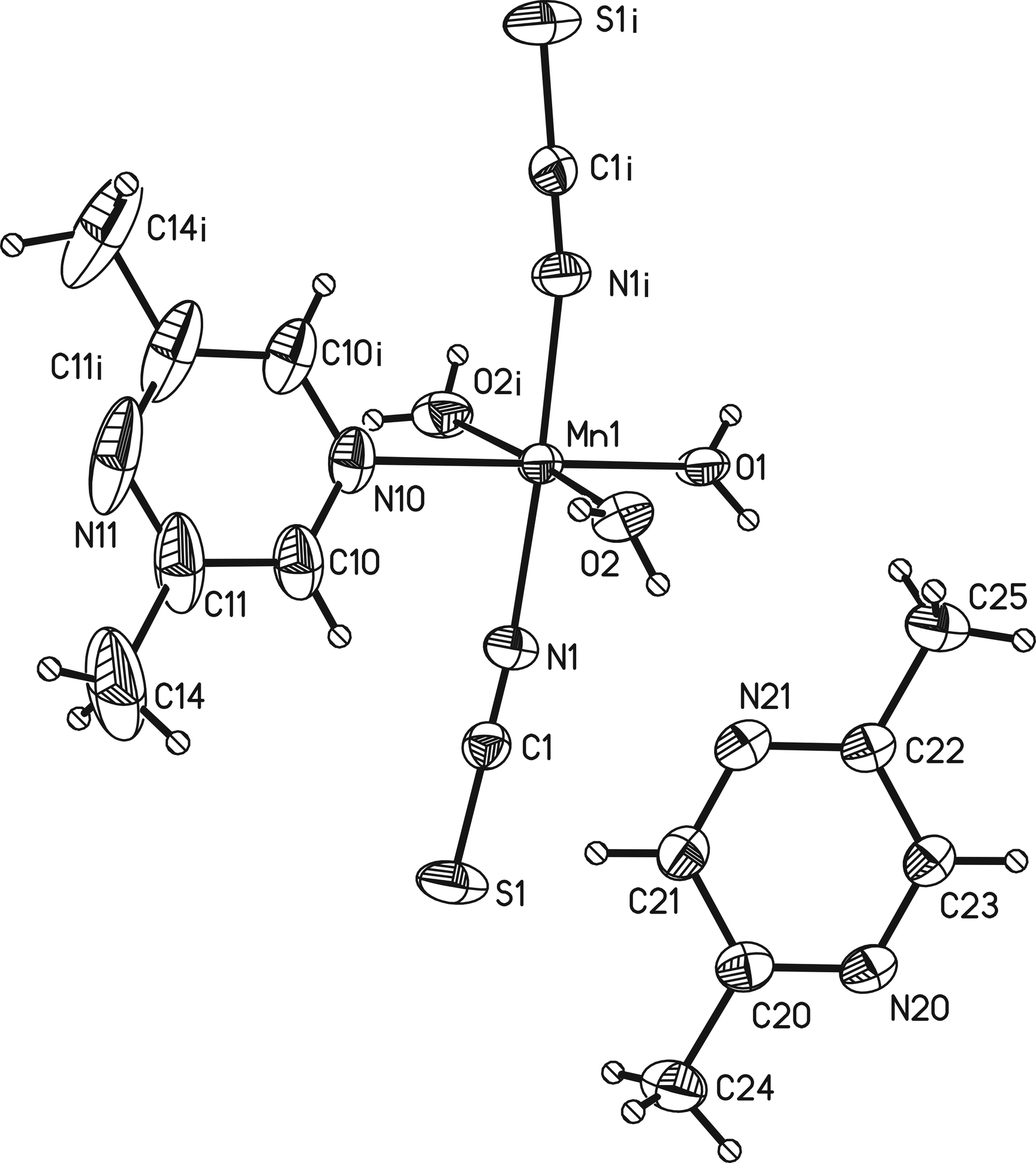

Supplement: Supplementary file 2 [file e-71-0m223-fig1.tif]

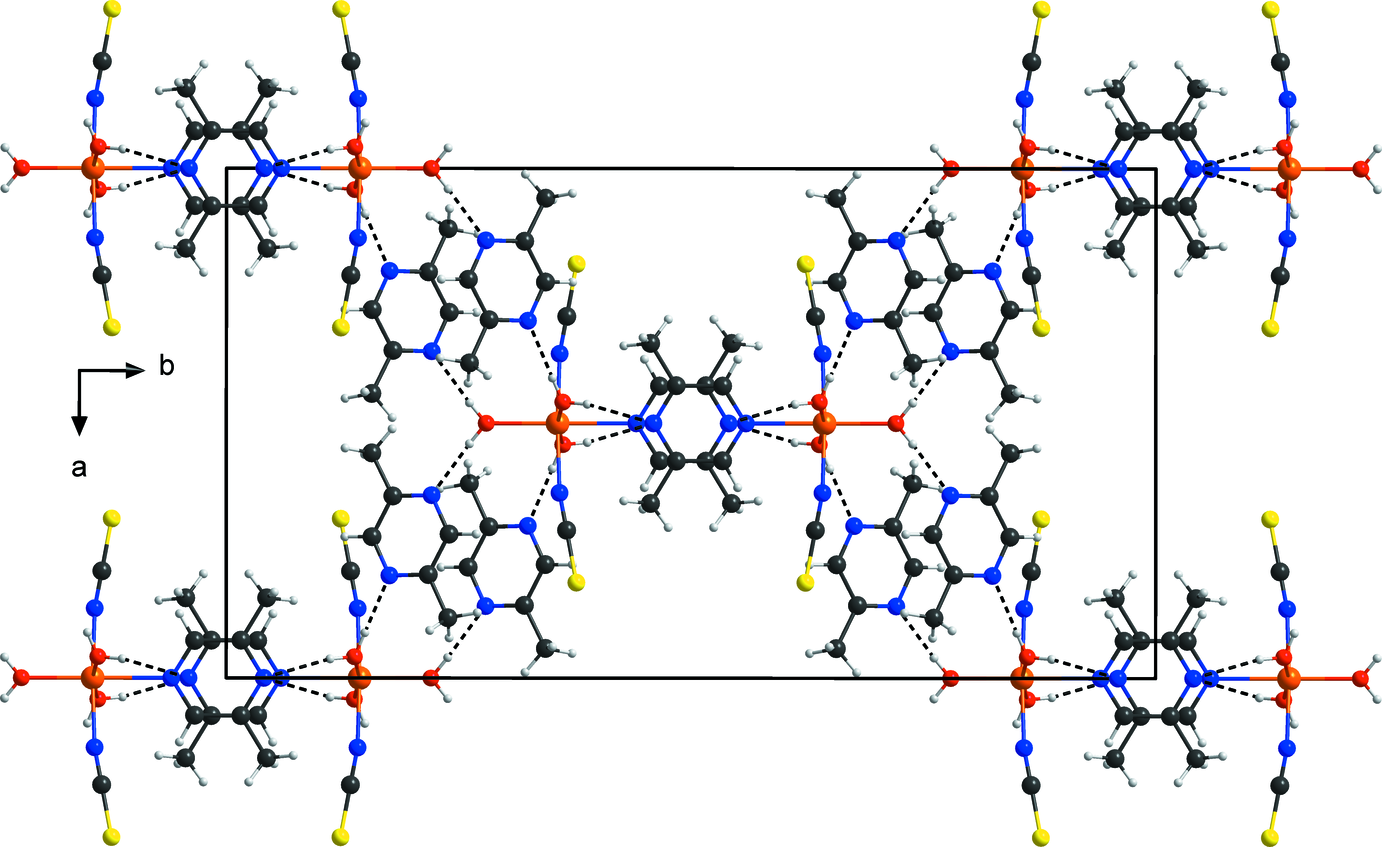

Supplement: Supplementary file 3 [file e-71-0m223-fig2.tif]
